# Non-Hookean large elastic deformation in bulk crystalline metals

**DOI:** 10.1038/s41467-022-32930-9

**Published:** 2022-09-27

**Authors:** Sheng Xu, Takumi Odaira, Shunsuke Sato, Xiao Xu, Toshihiro Omori, Stefanus Harjo, Takuro Kawasaki, Hanuš Seiner, Kristýna Zoubková, Yasukazu Murakami, Ryosuke Kainuma

**Affiliations:** 1 Department of Materials Science, Graduate School of Engineering, Tohoku University, 6-6-02 Aoba-yama, Sendai, 980-8579 Japan; 2 J-PARC Center, Japan Atomic Energy Agency, Tokai, Ibaraki 319-1195 Japan; 3 Institue of Thermomechanics, Czech Academy of Sciences, Dolejškova 5, 182 00 Prague, Czech Republic; 4 Faculty of Nuclear Sciences and Physical Engineering, Czech Technical University in Prague, Trojanova 13, 112 00 Prague, Czech Republic; 5 Department of Applied Quantum Physics and Nuclear Engineering, Kyushu University, Fukuoka, 819-0395 Japan

**Keywords:** Metals and alloys, Mechanical properties

## Abstract

Crystalline metals can have large theoretical elastic strain limits. However, a macroscopic block of conventional crystalline metals practically suffers a very limited elastic deformation of <0.5% with a linear stress–strain relationship obeying Hooke’s law. Here, we report on the experimental observation of a large tensile elastic deformation with an elastic strain of >4.3% in a Cu-based single crystalline alloy at its bulk scale at room temperature. The large macroscopic elastic strain that originates from the reversible lattice strain of a single phase is demonstrated by in situ microstructure and neutron diffraction observations. Furthermore, the elastic reversible deformation, which is nonhysteretic and quasilinear, is associated with a pronounced elastic softening phenomenon. The increase in the stress gives rise to a reduced Young’s modulus, unlike the traditional Hooke’s law behaviour. The experimental discovery of a non-Hookean large elastic deformation offers the potential for the development of bulk crystalline metals as high-performance mechanical springs or for new applications via “elastic strain engineering.”

## Abstract (web summary)

Engineering metals often suffer from a small elastic deformation with a linear stress-strain relationship obeying Hooke’s law. Here the authors observe a large nonlinear tensile elastic deformation with a strain of >4.3% in a bulk Cu alloy that offers potential for elastic strain engineering.

## Introduction

The familiar metals and alloys in our daily lives are mostly crystalline materials, and they elastically deform because of temporary stretching or contracting of bonds between atoms^1,2^. The ideal elastic strain for a crystalline metal is the strain at which the lattice itself disintegrates and hence sets a firm upper bound of the elastic strain of the material^3^. Theoretically, this strain value can be in the order of 10% for most crystalline metals with absolutely no defects^4–6^. However, the bulk shape of crystalline metals, which is the state mostly used for practical engineering applications, generally suffers a very limited elastic strain of <0.5% because of the occurrence of inelastic relaxation mediated by discrete dislocations^7^, deformation twinning^8^, and stress-induced phase transformations^9^. The stress–strain relationship of bulk crystalline metals in the very small elastic regime is generally linear, thus obeying the well-known Hooke’s law^10^.

Metals with a large elastic deformation can be used as components of mechanical vibrational systems, sporting goods, and medical devices because of their large reversible strains and mechanical energy storage^11,12^. One approach to achieving a huge elastic deformation in crystalline metals is the downscaling method. When reducing the size of the sample to micro- or nanoscales, such as that of freestanding whiskers and nanowires, certain metals can demonstrate ultra-high yield strength and elastic strains of 4%–7%, thus approaching their theoretical values because of a dislocation starvation mechanism^13–16^. However, when crystalline metals are scaled up to bulk sizes, the realization of a large elastic deformation has been confirmed to be challenging. It has been only reported in certain Ti-based alloys previously, such as the so-called gum metals, which are now used in medical and dental equipment. The elastic strain at room temperature of Ti-based gum metals can be as large as 2.5% with a Young’s modulus of ~60 GPa when the material is severely cold worked, probably because of a dislocation-free deformation mode^17^. Recent research also demonstrates that a large compressive elastic strain of ~2% as well as a Young’s modulus of ~100 GPa can be achieved in bulk chemically complex (or high-entropy) alloys because of the existence of localized high lattice distortion^18^. Note that the macroscopic elastic strain should correspond to the reversible lattice strain of a single phase for a true elastic deformation. On the other hand, certain shape memory alloys may exhibit elastic-like strains of >2% with slim stress hystereses as well as reduced apparent Young’s moduli, which are, however, associated with continuous or localized martensitic phase transition; hence such a deformation is pseudoelastic rather than truly elastic^19–22^. For example, the single-crystalline Ni–Co–Fe–Ga alloy fibers show a non-hysteretic recoverable strain of >15% and an incipient apparent Young’s modulus of ~25 GPa due to the continuous transformation from an ordered body-centered cubic (BCC) parent phase (B2) to a tetragonal martensite phase (L1_0_)^20,23^. The nanocrystalline Ni-Ti alloys may also show an elastic-like strain of >3% and an apparent Young’s modulus of ~35 GPa because of the reversible nanoscale B2 → B19’ phase transformation^21^. Here, to stabilize the localized martensite phase, sometimes pre-straining as a training procedure is even required^21,22,24^.

As seen from Hooke’s law, to achieve a large yet true elastic strain in bulk metals, both a low Young’s modulus and high strength are required. However, there is usually a trade-off between these properties for conventional metallic materials. For example, although having a low Young’s modulus of ~45 GPa, Mg alloys are not sufficiently strong to be elastically deformed to a large extent^25^. On the contrary, certain maraging steels can be very strong without yielding at stresses of >1 GPa, but they are extremely stiff with a Young’s modulus of >200 GPa to tolerate a larger elastic deformation^26^. We noticed that in a series of Cu–Al-based alloys, low Young’s moduli along the <100> orientation of a cubic-structured crystal can be identified^27–29^. A typical example is the Cu–Al–Ni alloy, which shows a very low Young’s modulus of ~25 GPa along the <100> crystal orientation, although a stress-induced martensitic phase transformation appears at relatively low elastic strains^27^. Cu–Al-based alloys also have ordered atomic structures in the parent phase; thus, a high yield strength can be expected because of the ordering strengthening effect^30^. We therefore speculate that a large elastic strain can be obtained in these alloys if the stress-induced phase transformation can be properly suppressed.

In this work, we report a large true elastic strain of >4.3%, which is experimentally determined by a tensile test at room temperature (~298 K), for a bulk and crystalline Cu–Al–Mn alloy. To date, this true tensile elastic strain of >4.3% represents the highest experimental value ever reported for bulk crystalline metals at room temperature. This bulk alloy also exhibits a nonlinear elastic response in stress–strain relationships, which does not obey the traditional Hooke’s law.

## Results

**Fig. 1:**
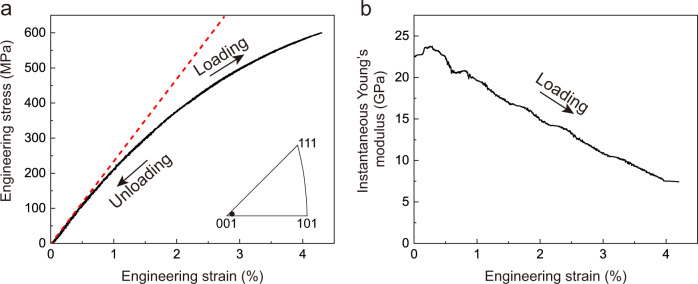
Elastic response of the bulk <100> Cu–Al–Mn single crystals. **a** Tensile engineering stress–strain curves of a near-<100> single crystal at room temperature with loading to 600 MPa and unloading to zero stress. The red dashed line is a guide representing linear elastic response. **b** The instantaneous Young’s modulus, i.e., $\frac{{{{{{\rm{d}}}}}}{{{{{\rm{\sigma }}}}}}}{{{{{{\rm{d}}}}}}{{{{{\rm{\varepsilon }}}}}}}$ (where *σ* is the engineering stress and ε is the engineering strain), as a function of engineering strain during the loading process as derived from **a**.

Cu–Al–Mn alloys as benchmark materials have been selected because of their excellent grain-coarsening ability^31^. Bulk single crystals of Cu_69_Al_17_Mn_14_ (in atomic percent) with an L2_1_-type ordered BCC structure (β phase) were prepared in the form of sheets of ~60 mm long by abnormal grain growth induced by cyclic heat treatment^31,32^. The chemical composition of our Cu–Al–Mn alloy was designed to suppress phase transformations or decomposition with the single β phase remaining thermally stable from a temperature of 390 K down to 3 K (Supplementary Fig. 1). We stretched a < 100> single crystal by incremental uniaxial cyclic loading and unloading tests. Figure 1a shows the engineering stress–strain curves for a maximum applied stress of 600 MPa on quasistatic loading and unloading. The strain was measured using a high-accuracy contactless video extensometer. It was found that the specimen completely recovered its original undeformed shape when the applied stress was released. The reversible strain can be as high as 4.31%, which is one order of magnitude higher than the practical elastic strain limit in most conventional bulk crystalline metals such as stainless steel. The crystal fractured at applied engineering stress of 612 MPa with a total engineering strain of 4.58%, and without being proceeded by significant plastic deformation (Supplementary Fig. 2). The large reversible strain is phenomenologically attributed to the extremely low Young’s modulus and moderately high yield strength. The incipient Young’s modulus of ~24 GPa along with <100> direction is consistent with the results calculated from the elastic constants determined by laser-ultrasonic measurements (Table 1 and Supplementary Fig. 3). Note that the stress hysteresis of the loading–unloading loop is zero, which drastically differs from that of pseudoelasticity involving first-order martensitic phase transformations^33^. The absence of a hysteresis loop indicates that virtually no work is dissipated during a loading–unloading cycle. A significant elastic softening phenomenon, in which the instantaneous Young’s modulus becomes smaller with increasing stress, was observed. As shown in Fig. 1b, the instantaneous Young’s modulus, i.e., the instantaneous stress–strain slope, decreases gradually from ~24 GPa at zero stress to 7.5 GPa at a stress of 600 MPa. This elastic softening phenomenon also contributes to the observed large reversible strain. Moreover, the alloy can sustain the stress at a large strain level for an extended duration without creep, indicating no occurrence of significant inelastic relaxation (Supplementary Fig. 4).

**Table 1:** Elastic constants for the present Cu–Al–Mn alloy experimentally determined at room temperature and some related elastic properties

*C*_11_ (GPa)	*C*_12_ (GPa)	*C*_44_ (GPa)	*C*′ (GPa)	*B* (GPa)	*C*′/*B*	*A*	*E*_<100>_ (GPa)	*ν*_<100>_
126.7	111.0	87.1	7.85	116.23	0.068	11.1	23.2	0.4670

**Fig. 2:**
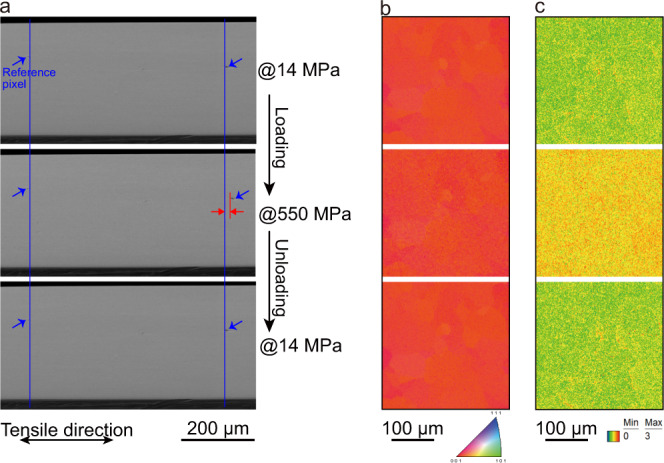
In situ microstructure observations during a tensile loading–unloading test. **a** SEM images showing the surface of the specimen before loading, loaded at 550 MPa, and after unloading. The blue arrows indicate the reference pixels. **b** The corresponding inverse pole figure maps and **c** the “Fit” value mapped in a cropped area obtained by using the EBSD technique.

That a localized stress-induced first-order martensitic phase transformation may cause similar mechanical responses in crystalline shape memory alloys is in some doubt^22^. To clarify the origin of the large reversible deformation or to confirm whether it is a true elastic deformation, we first conducted in situ microstructure observations during the tensile tests using a scanning electron microscope (SEM). Because of the dog-bone-shaped geometry and single crystal nature of the specimen, a uniform strain during the tensile deformation should be extensively spread. Figure 2a shows typical micrographs obtained before loading, after loading, and after unloading the tensile specimen. Surface relief, which is usually a typical symbol of a first-order martensitic phase transformation^33^, was not detected during the tensile deformation. The total reversible strain calibrated using the image pixels at an applied stress of 550 MPa was 3.4%, which is in good agreement with the value obtained from Fig. 1a. The electron-backscattered diffraction (EBSD) technique was employed to characterize the microstructure. Figure 2b shows the inverse pole figure maps along with the tensile directions. The phases could be identified to have a BCC structure in which the crystal orientation did not deviate during the deformation. A “Fit” parameter defined as the average angular deviation between the calculated Kikuchi bands and the detected Kikuchi bands in the EBSD measurement is shown in Fig. 2c. It can be seen that it increases because of the distortion of BCC lattices during loading and then drops back after unloading. These in situ microstructure observations show that neither martensitic phase transformation nor twinning takes place during the large reversible deformation. The absence of phase transformation and twinning during the deformation was also validated through observations using an in situ transmission electron microscope (TEM) at the nanoscale (Supplementary Fig. 5). Moreover, unlike the elastic-like or pseudoelastic behaviors of certain shape memory alloys undergoing weak first-order (overcritical) transitions or continuous transitions because of defects^19,20,34^, no inflection points or other deviation from monotonicity are observed along the stress–strain curve. Hence, the large reversible deformation in the present bulk Cu–Al–Mn alloy is presumably an intrinsic elastic property of the single β phase.

**Fig. 3:**
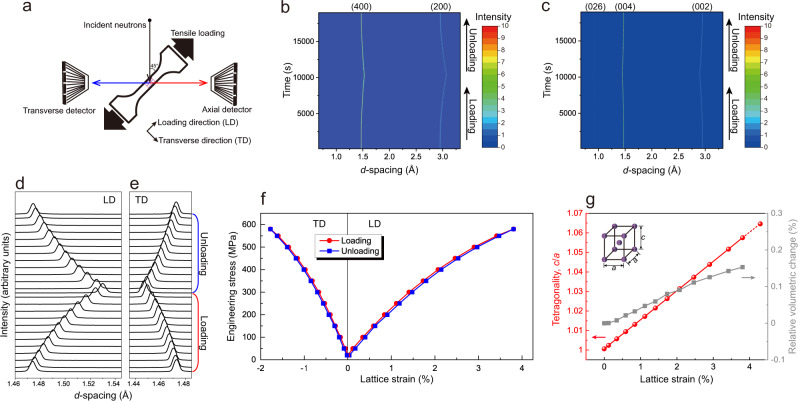
Microscopic responses revealed by in situ neutron diffraction. **a** In situ neutron diffraction measurement setup. LD loading direction, TD transverse direction. Two-dimensional neutron diffraction patterns collected during loading and unloading in the **b** LD and **c** TD. Evolution of the {400} diffraction peaks during loading and unloading in the **d** LD and **e** TD. **f** The tensile engineering stress as a function of the *d*-spacing strains for the (400) and (004) planes during loading and unloading. **g** The tetragonality of the cubic crystal and the relative volumetric change as a function of (400) *d*-spacing strains.

To gain further insight into the anomalous deformation behavior, in situ neutron diffraction measurements were performed on a single crystal stretched along the <100> direction at room temperature. The experimental configuration is schematically shown in Fig. 3a. Using the time-of-flight method, neutron diffraction patterns in the loading direction (LD) and transverse direction (TD) of the tensile specimen at different stress levels were simultaneously collected. Panels b and c in Fig. 3 show the plane view of neutron diffraction profiles during loading and unloading in the LD and TD, respectively. A maximum applied stress of 580 MPa was selected so as not to damage the specimen. The diffraction patterns shift continuously and reversibly on applying and releasing the stresses in both the LD and TD. For *d*-spacing ranging from 0.67 to 3.33 Å, no additional lines were detected during the measurement, indicating the absence of phase transformations or decomposition during the reversible deformation, in agreement with the results obtained for in situ microstructure observations.

To quantitatively evaluate the structural changes on the atomic scale, the one-dimensional diffraction patterns corresponding to the (400) reflection peak in the LD and (004) reflection peak in the TD were analyzed during loading and unloading, as shown in panels d and e in Fig. 3, respectively. The peaks shift continuously and reversibly, whereas no other new peaks can be identified at higher stresses. The direction of the peak shift for the TD is opposite to that in the LD, indicating shrinkage in the TD and an extension in the LD. The lattice strains, which refer to a relative change of the interplanar distance in comparison with its initial value before loading in the single β phase, are plotted against applied engineering stress in Fig. 3f. One can see that the engineering stress–lattice strain curve in the LD has a similar shape to that of the macroscopic engineering stress–strain curve shown in Fig. 1a. The maximum reversible lattice strain in the LD at an applied stress of 580 MPa is 3.81%, which is extremely large and in good agreement with the macroscopically measured engineering strain of 3.83% (Supplementary Fig. 6). Poisson’s ratio, defined as the negative ratio between TD and LD lattice strains, reaches a value of 0.4665. A very similar value of the Poisson’s ratio (*ν* = 0.4670) can be also calculated using the experimentally determined elastic constants (Table 1). This value is close to 0.5, a Poisson’s ratio for ideal rubber materials, indicating that the present crystal shows a rubber-like mechanical behavior in which the shape easily changes while volume tends to remain invariant during the tensile deformation^35^. The relative volumetric change at a lattice strain of 3.81% is very small, ~0.15%, as shown in Fig. 3g. Furthermore, at a high elastic strain prior to fracture, the initial cubic crystal (lattice constant *a*_0_ = 5.8955 Å for an L2_1_-type cell) becomes tetragonal with a huge tetragonality *c*/*a* of 1.065 where *c* and *a* are the lattice parameters for the *c* and *a* axes, respectively. Such a large tetragonality on elastic deformation has been rarely observed in cubic structured crystalline metallic materials. The abovementioned results obtained by in situ neutron diffraction measurements demonstrate that the large reversible tensile deformation at room temperature in the present alloy is truly an elastic deformation of the single β phase. This conclusion is supported by the observation of thermoelastic effect, in which a typical thermal phenomenon is induced by a true elastic deformation^36^, in the present alloy (Supplementary Fig. 7).

## Discussion

The low Young’s modulus and moderately high strength, which give rise to the large elastic deformation, are remarkable. The present alloy has an incipient Young’s modulus smaller than even that of Mg alloys (~45 GPa) but with apparently higher strength. We consider the low $[110](1\bar{1}0)$ shear modulus *C*′ of the cubic crystal to be important for that very low Young’s modulus. The value of *C*′ = 7.85 GPa in the present alloy is extremely small and has a direct contribution to the low <100> Young’s modulus when the bulk modulus representing the resistance to hydrostatic deformation is large (Table 1)^37^. This small *C*′ may be associated with the lattice instability of the β phase, which implies a low energy barrier for structural change in the tetragonal path to another structure during deformation^38–40^. Once the premature occurrence of such a structural change, usually a first-order martensitic phase transformation, is suppressed (this is exactly what we did for our alloy by tailoring the composition), the lattice may be highly distorted to yield a huge elastic deformation as well as a significant lattice anharmonicity^38,39,41^. The strong lattice anharmonicity induces the elastic softening upon tension, which results in a non-Hookean elastic response (see details in Supplementary Discussion, Supplementary Figs. 8–10). Note that the large elastic deformation and elastic softening phenomenon become weak or absent for stretching along the <110> or <111> direction because that *C*′ is less involved in those cases (Supplementary Fig. 11). The lattice anharmonicity of the β phase has a weak temperature dependence (Supplementary Fig. 13), and its existence is further demonstrated by the observation of an elastic stiffening phenomenon on uniaxial compression (see details in Supplementary Discussion, Supplementary Figs. 14–15). On the other hand, the moderately high strength is considered to result from the atomic ordering of the L2_1_ structure in the single β phase where dislocations related to plastic deformation are difficult to generate and migrate because of the involvement of antiphase–phase boundary (APB) energies^30,42^. Typically, the average APB energy for a Heusler Cu_2_MnAl alloy is reported to be extremely high (503 mJ m^−2^), indicating a high resistance to plastic deformation^43^. Although the yield strength of 612 MPa in the <100> deformation in the present alloy is slightly smaller than that of 679 MPa for a full Heusler structure at room temperature^42^, which is presumably caused by the lower degree of atomic ordering in an off-stoichiometric composition (Supplementary Fig. 12). The large tensile elastic deformation may be discovered in multiple other bulk BCC crystalline metals with similar features to the present alloy such as the low tetragonal shear resistance for a weak resistance to crystal distortion and the long-range atomic ordered single phase for high resistance to dislocation gliding (see details in Supplementary Discussion).

**Fig. 4:**
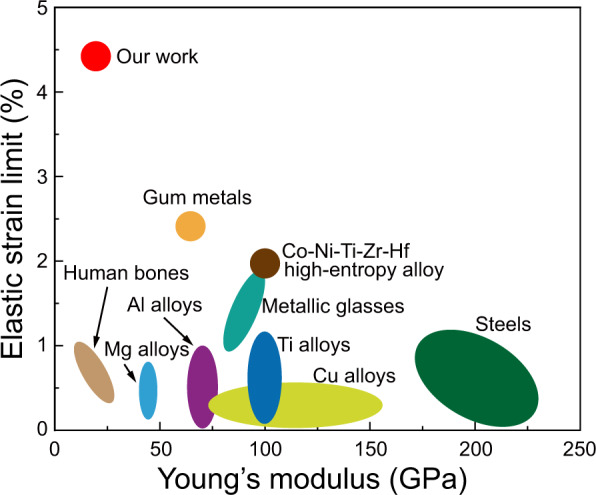
Comparison of elastic strain limit and Young’s modulus for various bulk metals and human bones. Note that the pseudoelastic materials involving stress-induced first-order phase transformation were not included due to their nonelastic nature.

The large elastic deformation with an extremely low Young’s modulus in bulk metals at room temperature is useful for multiple potential applications. Figure 4 gives a comparison of elastic strain limit and Young’s modulus for various bulk metals, including amorphous and ultraelastic high-entropy alloys, and human bones. The metals showing pseudoelasticity are excluded because pseudoelasticity is a phase-transformation-induced shape change rather than a true elasticity. The huge elastic strain experimentally obtained in this study is the highest among bulk metals. Because of the large elastic strain and the moderately high strength, the storage of a large amount of mechanical energy is enabled. The mechanical energy stored with applied stress of 600 MPa is 13.5 MJ m^−3^, which is comparable to that of bulk amorphous alloys (~15 MJ m^−3^) and much higher than that of commercialized spring steels (~1 MJ m^−3^)^11,44^. This feature is fascinating for multiple engineering applications such as in high-performance springs, self-locking joints, and pressure gauges. The low Young’s modulus very close to that of human bones (10–30 GPa) has potential applications in medical devices^12^. Furthermore, the point-to-point correspondence between stress (or strain) and apparent Young’s modulus because of the elastic softening phenomenon make the alloy available for applications requiring different material stiffness at certain stress (or strain) ranges. The ease of large-scale production of bulk single crystals with a controllable orientation by cyclic heat treatment and the low cost make the present alloy particularly advantageous for these applications^31,32,45^.

Recently, a growing interest has emerged in “elastic strain engineering,” in which lattice strains are engineered as a dynamic continuous variable for manipulating multiple physical or chemical properties of materials^46,47^. However, the elastic strain engineering applications at present are limited in nanostructures because bulk crystalline metals rarely sustain a sufficiently large elastic deformation^48,49^. The large elastic strains achieved in bulk metals, such as the present Cu–Al–Mn alloy, have the potential to use elastic strain engineering for emerging technologies beyond nanoscales such as in large-volume strain-mediated sensors^50–52^.

## Methods

### Specimen preparation

Ingots of Cu_69_Al_17_Mn_14_ were prepared by induction melting in an argon atmosphere. Then, the ingots were remelted and subjected to unidirectional solidification so that columnar polycrystalline structures with strong <100> textures along the solidification direction could be obtained. Sheet specimens with dimensions of approximately 60 mm × 10 mm × 1 mm were cut out using electrodischarging machining (EDM) so that the length will be parallel to the direction of solidification. Then, the sheet specimens were sandwiched between Mo sheets and subjected to a cyclic heat treatment. In this manner, large <100> single crystal sheet specimens could be obtained^45^. Additionally, sheet specimens cut from the noncolumnar area of the ingots were subjected to cyclic heat treatment to obtain large single crystals with other orientations along the length direction.

### Mechanical tests

To determine the limit of the large elastic deformation, a dog-bone-shaped specimen (15 mm × 4 mm × 1 mm in gauge dimension) with its tensile direction close to the <100> orientation was stretched to fracture by incremental cyclic loading–unloading tests using a tensile tester (Shimadzu AG-X 10 kN). At room temperature, the specimen was initially loaded to a stress of 550 MPa and then unloaded. This was followed by reloading to a stress of 600 MPa in the second cycle, and so forth. The strains were measured using a noncontact video extensometer (Shimadzu TRViewX240S) with high accuracy. The strain rate was 5 × 10^−4^ s^−1^.

To directly evaluate the anisotropic elastic properties, quasistatic tensile tests at room temperature were also conducted for <110> and <111> bulk single crystals. Dog-bone-shaped specimens with a gauge dimension of 15 mm × 4 mm × 1 mm were cut out from the single-crystal sheets using EDM. The crystal orientations of the specimens were determined using the EBSD technique. The strains were carefully measured using strain gauges (Kyowa KFGS-2-120-C1-11) attached onto the surface of the specimens. A loading–unloading cycle with a strain rate of 5 × 10^−4^ s^−1^ was conducted for all specimens. The maximum applied stress was set to 550 MPa so as not to damage the specimens.

In the engineering stress–strain curves, engineering stress σ is defined as the ratio of the applied force *F* to the initial cross-sectional area *A*_0_ of the tensile specimen, $\sigma=\frac{F}{{A}_{0}}.$ Engineering strain *ε* is defined as the ratio of extension Δ*L* = *L* − *L*_0_ to the initial gauge length *L*_0_ of the tensile specimen, $\varepsilon=\frac{\triangle L}{{L}_{0}}$.

The uniaxial compression test at room temperature was conducted on a bulk <100> Cu–Al–Mn single crystal. A rectangular parallelepiped specimen with a dimension of 7.41 mm × 3.00 mm × 2.95 mm was used. The specimen was loaded to a stress of 600 MPa and then unloaded. The strain was measured by strain gauges (Kyowa KFGS-2-120-C1-11) attached onto the surface of the specimen. The strain rate was 5 × 10^−4^ s^−1^.

The three-point bending tests were also performed on the present Cu–Al–Mn single crystal sheet at room temperature. The sheet was 40 mm × 3.86 mm × 0.30 mm in dimension with its longitudinal direction paralleling to <100> orientation of the crystal. The support span was 10 mm, and the crosshead speed was set at 1 mm min^−1^. The tests were conducted by a step-wise increasing loading–unloading sequence where the crosshead displacement interval step was set as 0.2 mm. The tests were finally finished by loading the specimen to 1.2 mm in crosshead displacement and unloading to a force of 0.2 N. The crosshead displacement was used to record the force–deflection curves. The same three-point bending tests were also conducted on a commercial Mo sheet purchased from the Nilaco Corporation as a comparison experiment.

### In situ microstructure observation

In situ microstructure observation during tensile loading and unloading was conducted using a field-emission scanning electron microscope (FE-SEM, Philips XL-3000) equipped with an EBSD detector. A tensile stage tilted to 70° was used at a working distance of 18.2 mm. The specimen was in a dog-bone shape and had a gauge dimension of 10 mm × 2 mm × 1 mm. The <100> orientation of the single crystal was close to the long axis of the specimen. At room temperature, the specimen was firstly prestretched using a 14 MPa stress, then further loaded to 500 MPa in steps of 100 MPa. It was then loaded to 550 MPa, which did not lead to the fracture of the specimen, and then finally unloaded. The crosshead speed was set at 0.4 mm min^−1^. The EBSD data were collected at each stress point and were analyzed using a TSL-OIM software.

In situ microstructure observation under tensile stresses was also conducted using a transmission electron microscope (TEM, JEOL JEM-3200FSK) and a single-tilt straining holder (Gatan Model-671). A single-crystal specimen with a near <100> orientation was used. The area of interest for observation was polished down to a thickness of about 100 μm, of which the electron transparency area was prepared by twin-jet polishing with a solution of 45% distilled water, 25% phosphoric acid, 25% ethanol, and 5% propanol at room temperature. Inside the electron microscope, the specimen was stretched to a maximum nominal strain of 5% with a step interval of 1%. The nominal strain is calculated as the ratio between the travel distance of the crosshead and the length of the specimen.

### In situ neutron diffraction measurements

In situ neutron diffraction experiments under tensile loading and unloading at room temperature were conducted using the Engineering Materials Diffractometer TAKUMI at the Materials and Life Science Facility (MLF) of Japan Proton Accelerator Research Complex (J-PARC). A single-crystal, dog-bone-shaped specimen with a gauge dimension of 20 mm × 3.4 mm × 1 mm was used. The specimen had a near [100] orientation along the loading direction and a near [001] orientation along the transverse direction. The loading machine was aligned with the load axis horizontal at an angle of 45° to the incident neutron beam. The time-of-flight neutron diffraction data were collected simultaneously for the loading and transverse directions by two detector banks at ±90° to the incident neutron beam. The data were continuously collected with a 600 s collection duration for holding stress states during both loading and unloading (this procedure also checked the stability of elastic deformation). The holding static stresses were set at 20, 50, 100, 150, 200, 250, 300, 350, 400, 450, 500, 550, and 580 MPa, respectively. An incident neutron beam slit of 5 mm × 5 mm with a pair of 5 mm radial collimators was used. The MLF beam power during the measurement was approximately 500 kW. The neutron diffraction data were analyzed using a Z-Rietveld software. During the measurement, the macroscopic strain of the specimen was measured using a clip-on extensometer (Epsilon Model-3442).

### Determination of elastic constants and elastic anisotropy

The elastic constants of the Cu_69_Al_17_Mn_14_ alloy were precisely evaluated at room temperature using the laser–ultrasonic transient grating spectroscopy method^53^. The measurements were performed on an approximately (110)-oriented single crystal. The alloy exhibits a very small tetragonal shear modulus (${C}^{{\prime} }=\frac{{C}_{11}-{C}_{12}}{2}$) of 7.85 GPa and therefore a large elastic anisotropy ($A=\frac{{C}_{44}}{{C}^{{\prime} }}$) of 11.1. In an anisotropic cubic crystal, the Young’s modulus *E* along a certain direction **n** (unit vector) is given by the following equation^54^:

1 $$\frac{1}{E({{{{{\bf{n}}}}}})}=\frac{1}{3({C}_{11}+2{C}_{12})}-\frac{1-3P\left({{{{{\bf{n}}}}}}\right)}{3\left({C}_{11}-{C}_{12}\right)}+\frac{1-P\left({{{{{\bf{n}}}}}}\right)}{2{C}_{44}},$$

where

2 $$P\left({{{{{\bf{n}}}}}}\right)={n}_{1}^{4}+{n}_{2}^{4}+{n}_{3}^{4}.$$

By using the experimentally determined elastic constants, the orientation dependence of Young’s modulus was calculated. The calculated incipient Young’s modulus along the <100> direction is 23.2 GPa, which agrees well with the value obtained from quasi-static tensile measurements in Fig. 1. The <100> Young’s modulus can also be written as a function of *C*′ and the bulk modulus ($B\,=\frac{{C}_{11}+2{C}_{12}}{3}$) as follows:

3 $${E}_{ < 100 > }=\frac{9{C}^{{\prime} }}{{C}^{{\prime} }/B+3}.$$

It can be seen that the low Young’s modulus along the <100> direction is mainly caused by the small *C*′ when *B* is far larger than *C*′. The Young’s modulus for the <110> and <111> directions were calculated using the same approaches. The results are *E*_<110>_ = 69.2 GPa and *E*_<111>_ = 209.1 GPa, respectively.

The Poisson’s ratio ν of a cubic crystal subjected to <100> uniaxial elastic deformation can be explicitly expressed in terms of elastic constants by^54^:

4 $${\nu }_{ < 100 > }=\frac{{C}_{12}}{{C}_{11}+{C}_{12}}=\frac{1}{2+\frac{{C}_{11}-{C}_{12}}{{C}_{12}}}=\frac{1}{2+\frac{2C^{\prime}}{{C}_{12}}}.$$

The calculated Poisson’s ratio is 0.4670. It is also seen that when the value of *C*′ is small, i.e., the value of $({C}_{11}-{C}_{12})$ approaching 0, the Poisson’s ratio in the <100> elastic deformation approaches 0.5.

### Evaluation of the thermoelastic effect

The thermoelastic effect describes the temperature change of a solid subjected to an adiabatic elastic deformation and is related to the pure volumetric deformation. Generally, under adiabatic conditions, elastic tension will cause a decrease in the specimen temperature, and elastic compression will lead to an increase in the specimen temperature^36^. The temperature change of a near-<100> Cu_69_Al_17_Mn_14_ single crystal under tensile loading and fast unloading was measured. Tensile loading–unloading tests at room temperature were conducted with a maximum stress of 550 MPa. The maximum elastic strain is then about 3.5%. The loading rate was 5 × 10^−2^ s^−1^, and the unloading rate was 1 × 10^−1^ s^−1^ to appropriate adiabatic conditions. A T-type thermocouple spot-welded to the central surface of the specimen was used to monitor the specimen temperature.

Theoretically, the adiabatic temperature changes of the specimen due to the thermoelastic effect in applying uniaxial stress can be given by the following equation^36^:

5 $$\triangle T=-\frac{{\alpha }_{{{{{{\rm{L}}}}}}}{T}_{0}}{{C}_{{{{{{\rm{p}}}}}}}\rho }\triangle \sigma,$$

where *α*_L_ is the coefficient of linear thermal expansion, *T*_0_ is the absolute temperature, Δ*σ* is the isentropic stress change, *C*_p_ is the specific heat at constant pressure, and *ρ* is the material density. For the present Cu–Al–Mn alloy, the values of these parameters are as follows: *α*_L_ = 1.29 × 10^−5^ K^−1^, *T*_0_ = 298 K, Δ*σ* = −550 MPa (unloading), *C*_p_ = 455 J kg^−1^ K^−1^, *ρ* = 7.40 × 10^3 ^kg m^−3^, respectively^55,56^. Subsequently, the temperature change Δ*T* during adiabatic unloading can be calculated to be +0.63 K. The experimentally obtained absolute value is +0.36 K (Supplementary Fig. 7), which is smaller than that calculated. This discrepancy may be caused by the nonideal adiabatic conditions of the experiments.

## Supplementary information

Supplementary Information

## Data Availability

The data that support the findings of this study are available from the corresponding authors upon request.
