# Supplementary material for: Non-Hookean large elastic deformation in bulk crystalline metals
Source: Nat Commun. 2022 Sep 27;13:5307. doi: 10.1038/s41467-022-32930-9 (PMC9515142; doi:10.1038/s41467-022-32930-9)
Supplement: Supplementary file 1 — Supplementary Information [file 41467_2022_32930_MOESM1_ESM.pdf]

Supplementary Information for

Non-Hookean large elastic deformation in bulk crystalline metals

Sheng Xu[1]*, Takumi Odaira[1], Shunsuke Sato[1], Xiao Xu[1], Toshihiro Omori[1], Stefanus Harjo[2], Takuro Kawasaki[2], Hanuš Seiner[3], Kristýna Zoubková[4], Yasukazu Murakami[5] & Ryosuke Kainuma[1]*

[1]Department of Materials Science, Graduate School of Engineering, Tohoku University, 6-6-02 Aoba-yama, Sendai 980-8579, Japan.

[2]J-PARC Center, Japan Atomic Energy Agency, Tokai, Ibaraki 319-1195, Japan.

[3]Institue of Thermomechanics, Czech Academy of Sciences, Dolejškova 5, 182 00 Prague, Czech Republic.

[4]Faculty of Nuclear Sciences and Physical Engineering, Czech Technical University in Prague, Trojanova 13, 112 00 Prague, Czech Republic.

[5]Department of Applied Quantum Physics and Nuclear Engineering, Kyushu University, Fukuoka 819-0395, Japan.

*Correspondence to: xu.sheng.a8@tohoku.ac.jp (S. Xu); kainuma@material.tohoku.ac.jp (R. Kainuma)

This file includes:

Supplementary Discussion 1-5

Supplementary Figures 1-15

Supplementary References

Supplementary Discussion

1. On the deviation from Hooke's law

The deviation from Hooke's law within large elastic deformation may have been experimentally observed in nanowires or whiskers due to lattice anharmonicity owing to the large deviations in interatomic separation from the equilibrium values[1], but it is not a frequent case for bulk crystalline metals in experiments. The linear elastic response of a crystalline material is represented by a quadratic term in the interatomic potential energy; however, nonlinear elasticity requires higher order terms[2]. The nonlinear large tensile elastic response in the present bulk Cu–Al–Mn single-crystalline alloy would suggest a high-order elastic behaviour, in which the stress–strain relationship for uniaxial loading can be expressed as follows[3]:

$$\sigma_{\mathrm{T}} = E\varepsilon_{\mathrm{T}} + D{\varepsilon_{\mathrm{T}}}^2 + \cdots \quad (1)$$

where σ_{T} is the true stress, which is the applied force F divided by the actual cross-sectional area A of the tensile specimen at that load, $\sigma_{\mathrm{T}} = \frac{F}{A}$; ε_{T} is the true strain, which is equal to the natural log of the quotient of current length L over the original length L_0, $\varepsilon_{\mathrm{T}} = \ln\frac{L}{L_0}$; and E and D are the incipient Young's modulus and third-order modulus, respectively. This relationship is valid for any dimensionality and has been used in evaluating large-strain elastic responses in nanostructures such as Pd nanowires and graphene membrane[3,4]. Our data for the tensile true stress–strain curves in the present bulk Cu–Al–Mn alloy shows that a quadratic fit describes the nonlinear response until fracture (Supplementary Fig. 8), where E and D are determined to be 23.3 and −202.8 GPa, respectively. The ratio between D and E ($b = D / E$), known as the strain-expanded nonlinearity parameter directly scaling the level of anharmonicity[3], was found to be −8.70. This large magnitude of nonlinearity parameter indicates a strong elastic softening upon tension beyond the linear region.

An elastic response implies the existence of a potential energy that is a function of strain. By considering Poisson's effect, a relationship between Equation (1) and the strain energy $U(\varepsilon_{\mathrm{T}})$ related to strain of a crystalline material with Poisson's ratio v and initial volume V_0 subjected to uniaxial tension can be appropriated as follows[3,5]:

$$\frac{U(\varepsilon_{\mathrm{T}})}{V_0} = E\left[\frac{1}{2}{\varepsilon_{\mathrm{T}}}^2 + \frac{1}{3}(1 - 2v + b){\varepsilon_{\mathrm{T}}}^3\right] \quad (2)$$

where the second-order term is the harmonic term, the third-order term represents the anharmonic contribution. Subsequently, a dimensionless expression for the fractional anharmonic contribution to the strain energy density as function of ε_{T} can be expressed as $\frac{2}{3}(1-2v+b)\varepsilon_{\mathrm{T}}$. Using v = 0.4665 (obtained from Fig. 3), this value would be −6% and −25% for tensile true strains of 1% and 4.3%, respectively, in the present case (Supplementary Fig. 9a), indicating that lattice anharmonicity plays a substantial role for the strong elastic softening upon uniaxial tension particularly at a large-strain deformation.

Following Milstein's work[6], the following relation is obtained as a lattice stability criterion for a cubic crystal:

$$K \equiv \frac{\partial^2 (\frac{U(\varepsilon_{\mathrm{T}})}{V_0})}{\partial {\varepsilon_{\mathrm{T}}}^2} = E[1+2(1-2v+b)\varepsilon_{\mathrm{T}}] > 0 \quad (3)$$

The β-BCC lattice becomes unstable to the strain at which the *K* vanishes. Supplementary Fig. 9b plots the strain energy density $\frac{U}{V_0}$ and force constant *K* as a function of tensile true strain for the present alloy upon <100> tension. The ideal elastic strain limit can be then estimated to be 5.79% at a strength of 669 MPa (true strain and true stress). The experimental results of 639 MPa (true stress) and 4.47% (true strain) greatly approach the ideal values, putting the present Cu–Al–Mn alloy into the "ultrastrength materials" catalogue. Here, the term "ultrastrength" was coined in 2009 to describe material components sustaining sample-wide stress level at a significant fraction (>1/10th) of its ideal strength or 1% elastic strain pervasively[7,8].

By using Equation (2), the strain energy density $\frac{U}{V_0}$ as a function of true strain in both tension and compression is plotted in Supplementary Fig. 9c. It is seen that there is an asymmetry in strain energy density between tension and compression because of the lattice anharmonicity.

2. Key factors for achieving large tensile elastic deformation

Supplementary Fig.10 compares the strain energy landscape of the present Cu–Al–Mn alloy to that of typical bulk BCC-structured metallic elements for uniaxially stretching along the <100> direction, including Fe, W, Mo and Nb[9,10,11]. One can see that the increase in strain energy due to straining is far smaller in the present Cu–Al–Mn alloy, indicating that a smaller applied stress could induce a large elastic strain within the lattice stability region. Incidentally, this mild

strain energy landscape is in consistent with the results of Heusler Cu_2MnAl crystals obtained by first-principles calculations[12,13], which indicates that the crystal could distort easily due to intrinsic weak interatomic bonds. The mild characteristics in energy landscape can also reflect a low value of C' modulus and a low Young's modulus in cubic crystals[14].

Nevertheless, the inhibition of plastic deformation is a key issue for achieving a high yield strength. It is traditionally considered that metals showing a low Young's modulus have a low strength, such as Sn alloys, Al alloys, and Mg alloys. Typically, for example, in β-Sn or its alloys, a low C' modulus or a low Young's modulus may be identified, indicating a mild energy landscape on straining, yet their elastic strain is smaller than 0.2% due to the very small yield strength related to the occurrence of dislocation-stimulated plastic deformation[15]. However, being different from those conventional soft metals, the present Cu–Al–Mn alloy is featured by its long-range ordered atomic structure (ordered BCC structure, $L2_1$)[16], which should give rise to a high resistance to dislocation creation or movement for plastic deformation due to ordering strengthening effects[17]. Ordering strengthening can be mainly considered to be related to the antiphase boundary (APB) energy. Because the creation of superlattice dislocations in ordered structures will form an APB interface, which means that the critical stress to generate and move the dislocations should be larger due to the involvement of APB energy. The high APB energy (503 mJ m^{-2}) featured in Cu_2MnAl system indicates a high yield strength to plastic deformation[18]. The reported experimental yield strength for a <100> Cu_2MnAl single crystal uniaxially deformed at room temperature is about 680 MPa[19]. Incidentally, this value matches the intrinsic strength of 669 MPa in the present alloy. This matching between actual strength and intrinsic strength means that the present Cu–Al–Mn crystal can actually be distorted to a large extent to achieve a large elastic strain without premature involvement of dislocation-stimulated plastic deformation. Some other factors, such as antiphase domain size and quench-in vacancies (Supplementary Fig.12), may also contribute to the ordering strengthening effect[17], which represent a future research topic for the present Cu–Al–Mn alloy. Hence, large elastic deformation can be obtained in bulk BCC crystals with low resistance to distortion (this can be reflected by a low shear modulus C' or a low Young's modulus) and a long-range ordered atomic structure for high yield strength.

3. On the difference between elasticity and pseudoelasticity

Reversible deformations caused by mechanical stresses in crystalline metals can be due to a true elasticity or a pseudoelasticity. A true elastic behaviour in crystalline metals is a macroscopic manifestation of atomic bonding and thus a result of bonds against extension or compression and distortion. The elastic strain should then correspond to the reversible lattice strain in the single phase. Being different from a true elasticity, pseudoelasticity can be attributed to several other mechanisms, such as the stress-induced martensitic transformation (also known as superelasticity in shape memory alloys)[20,21], the twinning-untwining behaviour[22], or the reversible glide of dislocations (in rare cases)[23,24]. The strain in pseudoelasticity is actually inelastic; however, it is reversible. A true elasticity is noticeable for its features such as the zero stress hysteresis and the monotonicity in stress–strain relations.

Considering the more possible confuses between a true elasticity and a pseudoelasticity induced by stress-induced martensitic transformation (superelasticity), here the evidence for distinguishing them is presented.

- Stress–strain characteristics: In superelasticity, the stress-strain curve is usually highly nonlinear with a hysteresis loop (e.g., Supplementary Fig. 13a), because stress-induced martensitic transformation usually occurs in an avalanche-like manner, leading to strain burst and large energy dissipation[20]. For some alloys exhibiting confined stress-induced martensitic transformation or continuous phase transformation due to defects, the stress hysteresis can be greatly reduced; however, the inflection points in the stress–strain curve would indicate the presence of a weak first-order phase transition[25,26,27]. For a true elasticity, since there is no occurrence of first-order phase transition, the stress–strain relation is monotonic without any stress hysteresis (Fig. 1a).
- Microstructure characteristics: In superelasticity, stress-induced martensitic transformation occurs with the creation of interface between parent phases and martensite phases, which presents the surface relief in the specimen[21]. In a true elasticity, the deformation is homogeneous across the single phase; therefore, no surface relief or trace appears (Fig. 2a).
- Phase identification characteristics: In superelasticity, due to the transformation from parent phase to martensite phase with a different crystal structure, there should be additional peaks or lines that appear in the X-ray or neutron diffraction patterns. Upon the deformation processing, the intensity for the peaks or lines of martensite phases increases whereas that of parent phase decreases. In a true elasticity, the single-phase nature ensures that no additional

peaks or lines appear. The positions of peaks or lines shift continuously due to the lattice parameter change of the single phase during elastic deformation, whereas the intensities remain almost constant (Fig. 3).

- Thermal characteristics: In superelasticity, so-called elastocaloric effect occurs due to the entropy change between parent and martensite phases. In most cases, the temperature of the specimen greatly increases (by ~10 K in Cu-based shape memory alloys) upon adiabatic loading due to the transformation from parent phase to martensite phase, whereas it decreases upon adiabatic unloading due to reverse phase transformation[28]. In a true elasticity, a thermoelastic effect occurs due to the pure volumetric deformation of a single phase. Generally, upon uniaxial adiabatic loading, elastic tension will cause a decrease (usually of less than 1 K) in the specimen temperature, and elastic compression will cause an increase in the specimen temperature (Supplementary Fig. 7) [29].

4. On the relationship to pretransitional phenomena in martensitic transformation

In shape memory crystalline alloys, there are some studies reporting a drastic reduction of C' modulus upon cooling of presumably homogeneous parent phase towards the martensitic transformation[30], known as pretransitional phenomena. The significant softening of certain acoustic phonons has also been reported in the cooling process of a parent phase towards martensitic transformation[30]. We performed tensile test on the present Cu–Al–Mn alloy at lower temperatures by using another near-<100> single crystal, and stress-induced martensitic transformation occurs due to the reduced relative lattice stability upon cooling (Supplementary Fig. 13a). It is seen in Supplementary Fig. 13b that the incipient Young's modulus of the parent phase is almost constant at various temperatures and that drastic change due to pretransitional softening behaviours is not detected even before stress-induced martensitic transformation. This demonstrates that the low Young's modulus and the huge elastic deformation in the present alloy originate intrinsically in the parent phase, but they are not directly related to the pretransitional phenomena.

5. Elastic responses on compression and bending

Supplementary Fig. 14a shows the engineering stress–strain curves in compression of the present <100> Cu–Al–Mn single crystal. It is seen that the Young's modulus increases gradually with the increase in stress (elastic stiffening), yet a large elastic strain of over 1.8% can still be reached at an applied engineering stress of 600 MPa. The instantaneous Young's modulus increases from ~24 GPa at a stress of 20 MPa to ~40 GPa at a stress of 600 MPa, as shown in Supplementary Fig. 14b. The asymmetric elastic behaviour, i.e., the elastic stiffening in compression and elastic softening in tension, is a result of the lattice anharmonicity described in Supplementary Fig. 9c.

Supplementary Fig. 15a shows the force–deflection curves in bending of the present <100> Cu–Al–Mn single crystal. It is the seen that the crystal could be bended to a large deflection and then spring back to its original shape after unloading, being different from the bending response of Mo sheets undergoing plastic deformation, as also indicated by Supplementary Fig. 15 b and c. Note that the drop in force on unloading from large deflections in the force–deflection curves should be caused by drift of contact points between specimen and supports. At a deflection of 1.2 mm, the flexural strain $\varepsilon_{\text{flex}}$ in the outer surface can be roughly estimated to be 2.16% using the following equation[31]:

$$\varepsilon_{\text{flex}} = \frac{6\delta t}{L^2} \tag{4}$$

where δ (= 1.2 mm) is the deflection of the center of the specimen, t (= 0.30 mm) is the specimen thickness and L (= 10 mm) is the support span. Further, it is seen from Supplementary Fig. 15a that the Cu–Al–Mn single crystal is much less stiff than Mo. The bending modulus E_{bend} of the present <100> Cu–Al–Mn single crystal can be calculated using the following equation[31]:

$$E_{\text{bend}} = \frac{L^3 m}{4bt} \tag{5}$$

where m (= 14.86 N mm^{-1}) is the slope of the initial straight-line portion of the force–deflection curve and b (= 3.86 mm) is the width of the specimen. The calculated bending modulus is small to be 32.1 GPa, being slightly different from the value of tensile Young's modulus, which is due to the different deformation mode between bending and tension.

Supplementary Figures

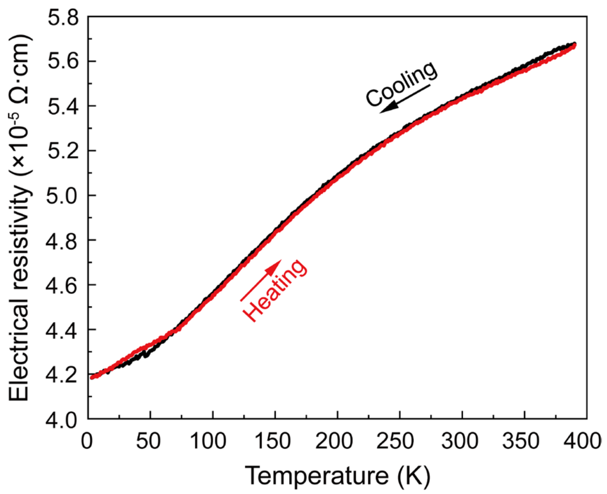

Supplementary Fig. 1 Electrical resistivity as a function of temperature. There is no abrupt change in electrical resistivity upon cooling, indicating a stable β phase.

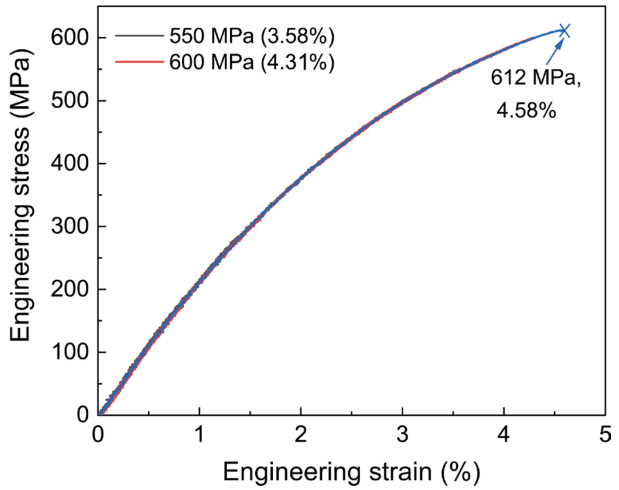

Supplementary Fig. 2 Stress–strain responses for tensile deformation to fracture. Engineering stress-strain curves up to fracture by incremental tensile loading–unloading tests at room temperature.

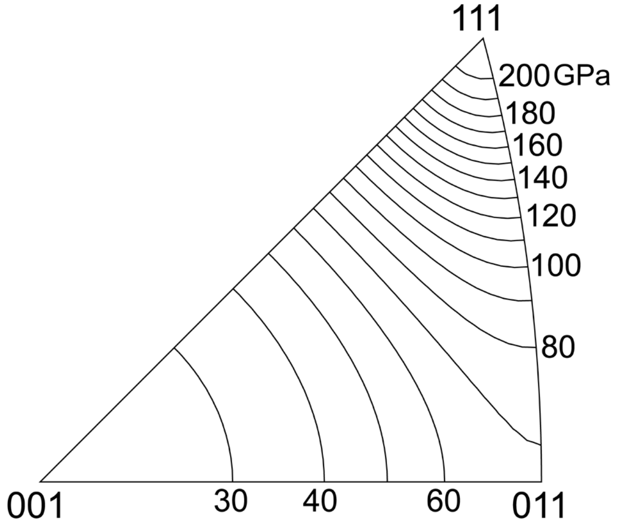

Supplementary Fig. 3 Orientation dependence of the incipient Young's modulus. The values are calculated using the experimentally determined elastic constants listed in Table 1. The values along the <001>, <011>, and <111> directions are 23.2, 69.2, and 209.1 GPa, respectively.

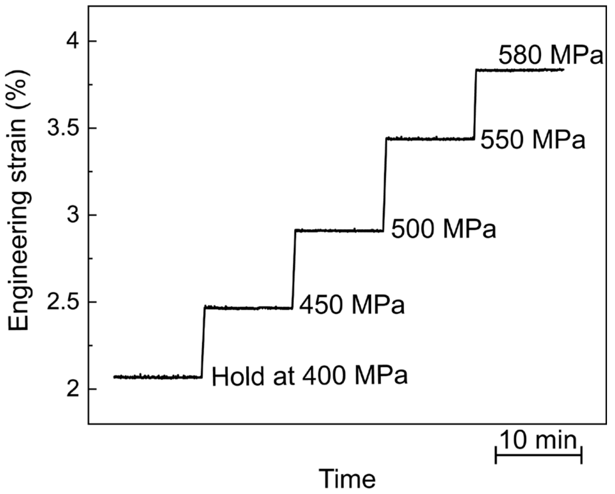

Supplementary Fig. 4 Stability of the large elastic deformation. The engineering strain is plotted as a function of time at several constant holding stresses. The elastic strain energy density can be stored without relaxation over a significant period.

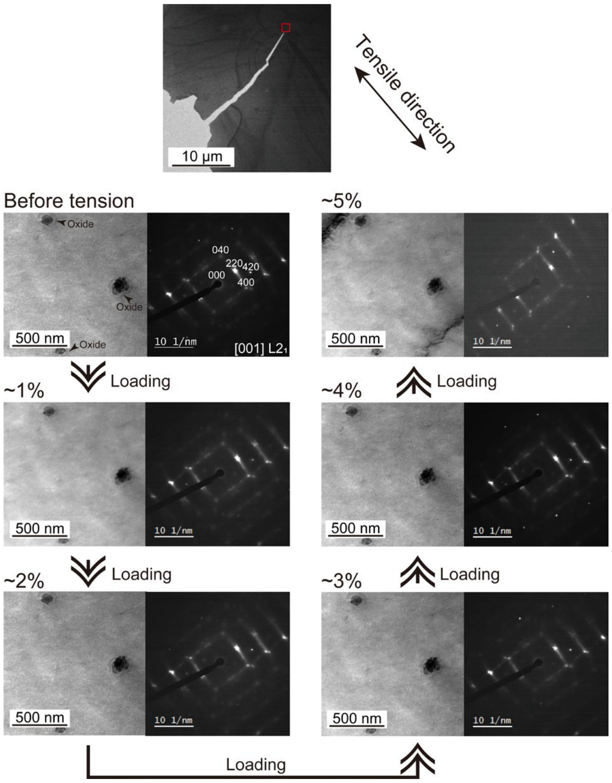

Supplementary Fig. 5 In situ microstructure observations inside a transmission electron microscope. The bright-field images in front of a crack tip and corresponding electron diffraction patterns at different nominal strain states during in situ tensile deformation at room temperature are presented. There is no phase transformation detected, as known from the invariance of electron diffraction patterns.

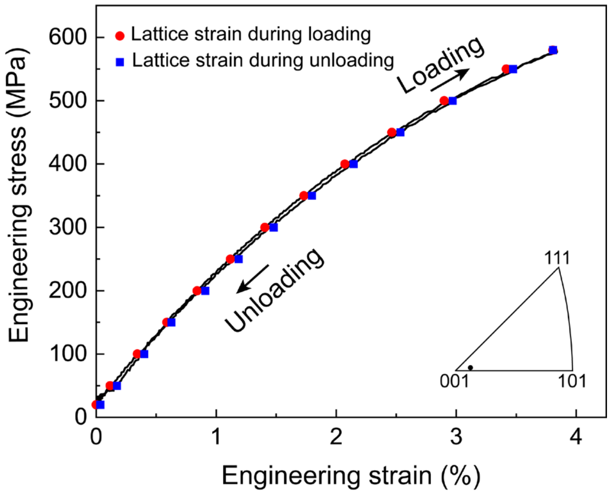

Supplementary Fig. 6 The observed macroscopic engineering stress–strain curves during the neutron diffraction measurement. The red and blue dots represent the corresponding {400} *d*-spacing strains in the direction of loading and unloading, respectively.

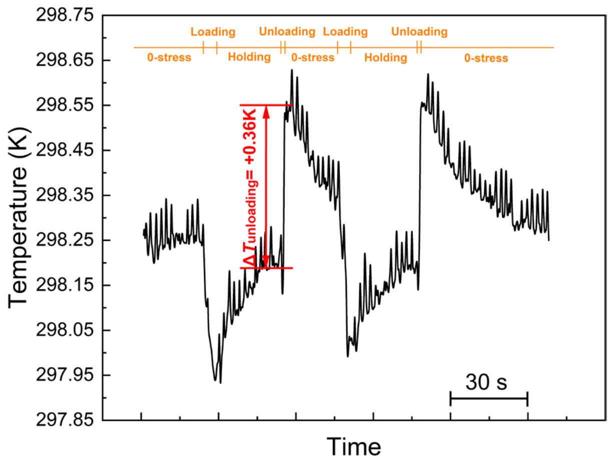

Supplementary Fig. 7 Thermoelastic effect due to the elastic tensile deformation. The specimen temperature was plotted as a function of time during tensile loading and fast unloading at room temperature with a maximum stress of 550 MPa. The specimen temperature increases by about 0.36 K during the adiabatically unloading process due to the thermoelastic effect induced by a true elastic deformation.

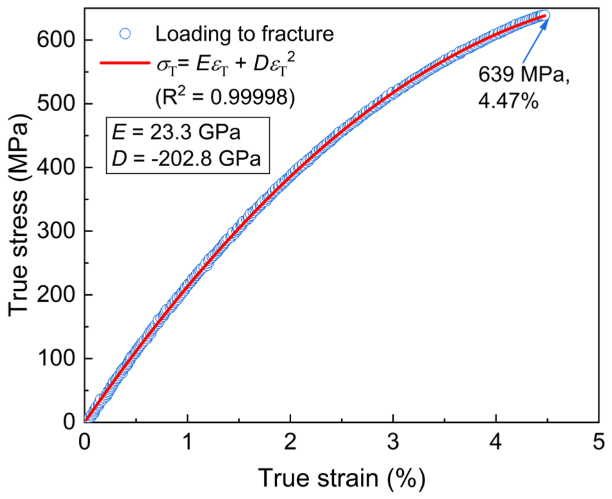

Supplementary Fig. 8 True stress–strain curves. The true stress–strain curves of loading to fracture and the fitted curves.

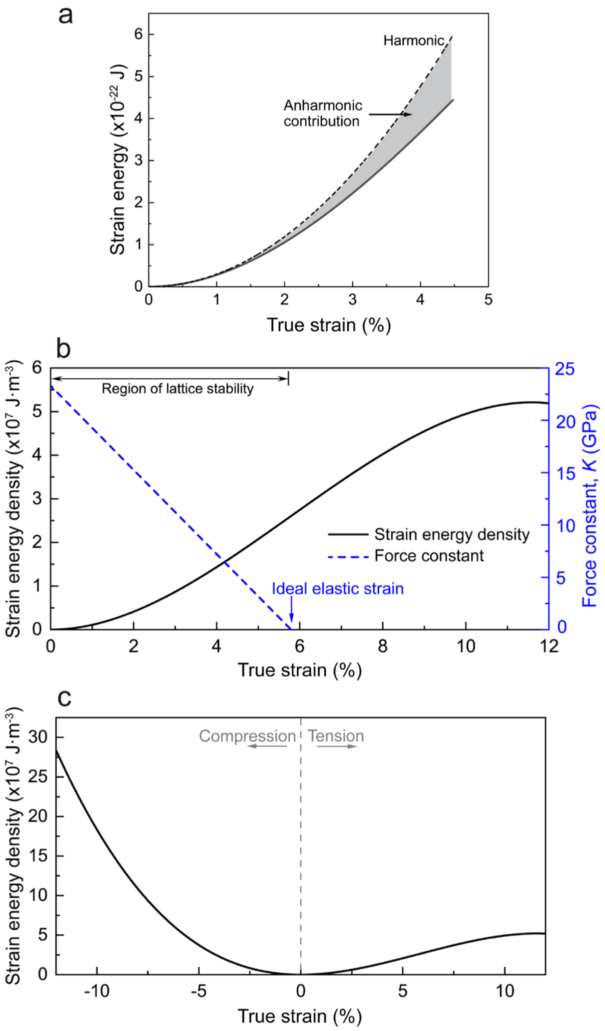

Supplementary Fig. 9 Energy landscape related to strain. (a) The strain energy U as a function of tensile true strain. The hatched area represents the anharmonic contribution to the strain energy. (b) The strain energy density $\frac{U}{V_0}$ and force constant K as a function of tensile true strain. (c) The strain energy density $\frac{U}{V_0}$ as a function of true strain in both tension and compression.

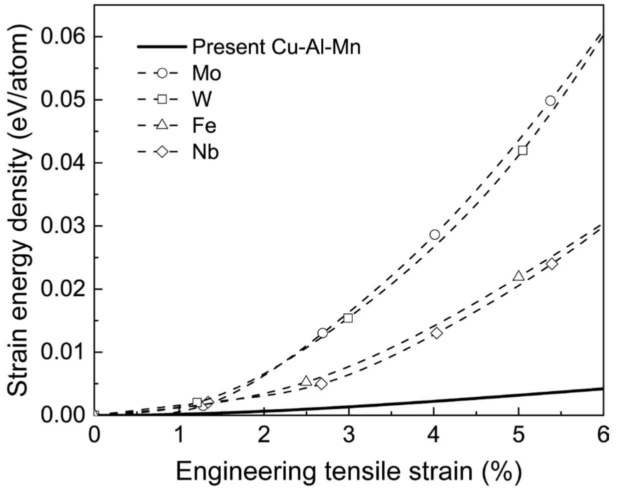

Supplementary Fig. 10 Comparison of energy landscape among the present Cu–Al–Mn and typical BCC elements. The data points for BCC elements were obtained by first-principles calculations[9,10,11].

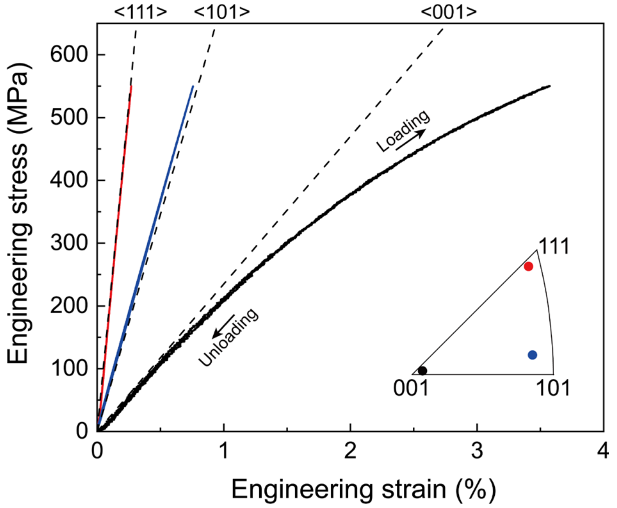

Supplementary Fig. 11 Anisotropic elastic responses. Anisotropic elastic properties during loading and unloading in tension on single crystals with an applied engineering stress of 550 MPa. The inset shows the crystal orientation of the specimen, which is parallel to the uniaxial loading direction. The dashed lines indicate the Young's modulus calculated using elastic constants in the <001>, <101>, and <111> directions.

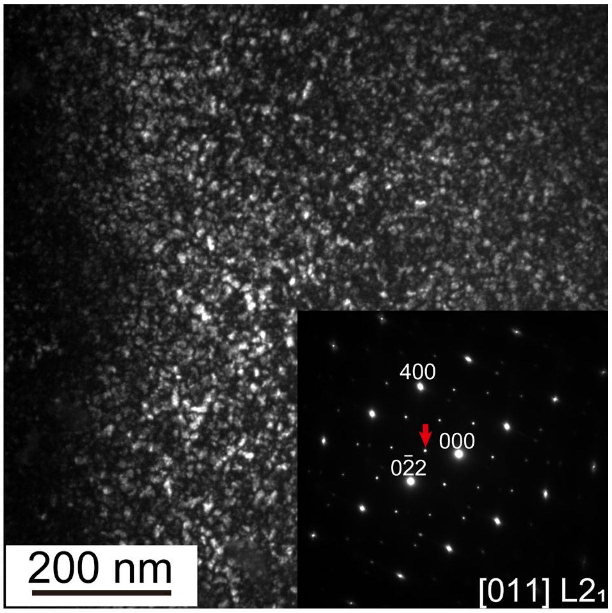

Supplementary Fig. 12 Microstructures at the nanoscale. Dark-filed TEM image taken from the $(1\bar{1}1)_{L2_1}$ superlattice reflection and the selected area electron diffraction pattern obtained with a [011] incident zone axis for the present Cu–Al–Mn alloy. The average size of ordered domains is about 8 nm in diameter.

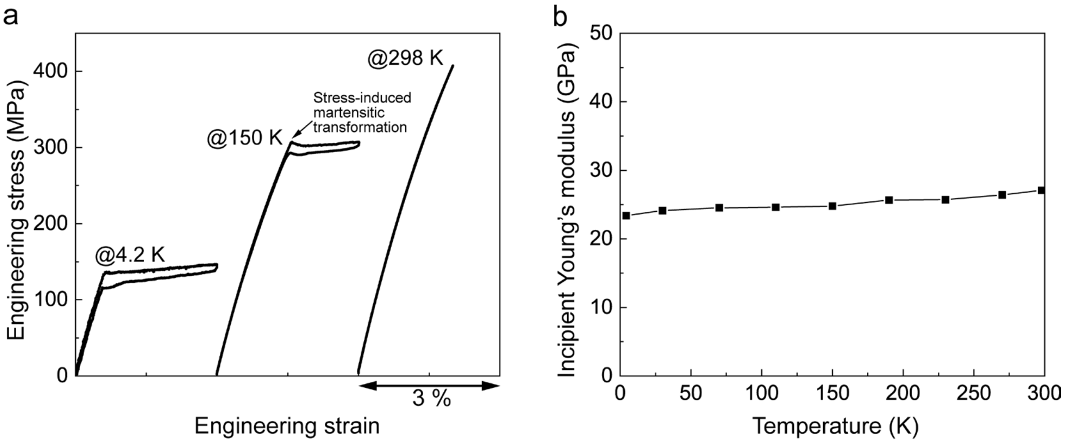

Supplementary Fig. 13 Tensile deformation behaviours at lower temperatures. (a) Tensile stress–strain curves at lower temperatures. Stress-induced martensitic transformation (SIMT) occurs at lower temperatures due to the reduced lattice stability of BCC phase. (b) The incipient Young's modulus as a function of testing temperature.

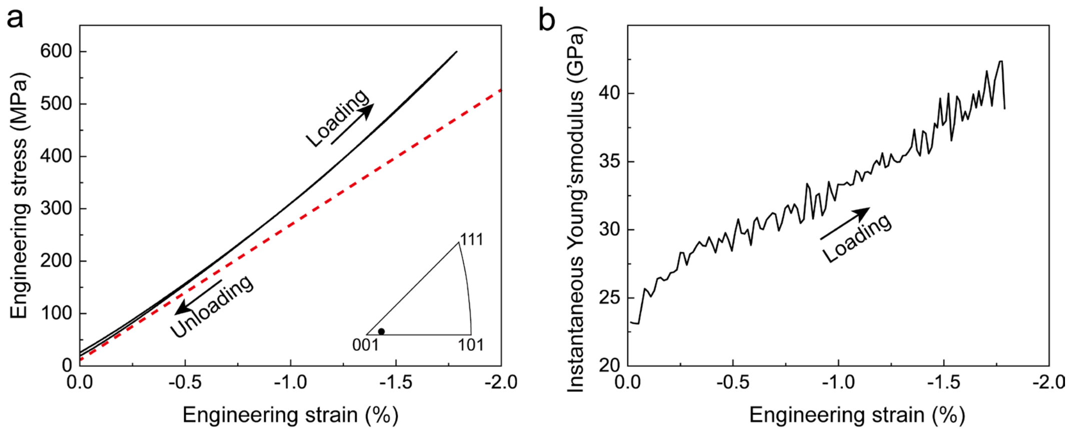

Supplementary Fig. 14 Elastic responses on compression. (a) Compressive engineering stress–strain curves of a near-<100> single crystal at room temperature with loading to 600 MPa and unloading to 20 MPa. The red dashed line is a guide representing linear elastic response. (b) The instantaneous Young's modulus as a function of engineering strain derived from the compressive loading engineering stress–strain curves.

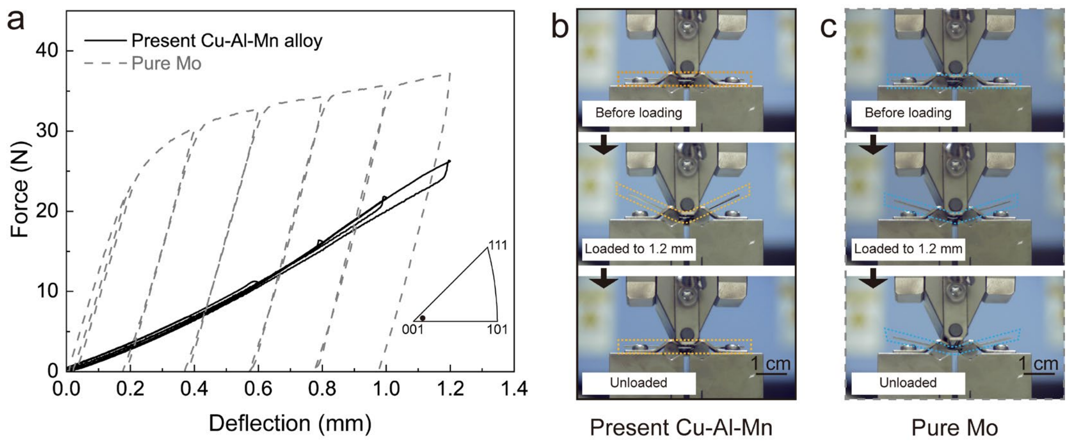

Supplementary Fig. 15 Elastic responses on bending. (a) Force–deflection curves obtained during the bending loading–unloading tests for a <100> Cu–Al–Mn single crystal, the inset shows the crystal orientation along the longitudinal direction of the specimen. The bending force–deflection curves of a Mo sheet specimen easily undergoing plastic deformation are plotted as dashed lines for comparison. The photographs captured during the bending tests for (b) the present Cu–Al–Mn single crystal and (c) the Mo sheet.

Supplementary References

1. Kobayashi, H. & Hiki, Y. Anharmonicity in noble metals; nonlinear elasticity in whiskers. *Phys. Rev. B* **7**: 594 (1973).
2. Landau, L. D. & Lifshitz, E. M. *Theory of elasticity.* Pergamon Press, London, 1959.
3. Chen, L. Y., Richter, G., Sullivan, J. P. & Gianola, D. S. Lattice anharmonicity in defect-free Pd nanowhiskers. *Phys. Rev. Lett*. **109**, 125503 (2012).
4. Lee, C., Wei, X., Kysar, J. W. & Hone, J. Measurement of the elastic properties and intrinsic strength of monolayer graphene. *Science* **321**, 385–388 (2008).
5. Diao, J., Gall, K., & Dunn, M. L. Atomistic simulation of the structure and elastic properties of gold nanowires. *J. Mech. Phys. Solids* **52**, 1935–1962 (2004).
6. Milstein, F., Theoretical strength of a perfect crystal. *Phys. Rev. B* **3**, 1130–1141 (1971).
7. Zhu, T., Li, J., Ogata, S. & Yip, S. Mechanics of ultra-strength materials. *MRS Bull.* **34**, 167–172 (2009) .
8. Zhu, T. & Li, J. Ultra-strength materials. *Prog. Mater. Sci.* **55**, 710–757 (2010).
9. Clatterbuck, D. M., Chrzan, D. C. & Morris Jr, J. W. The inherent tensile strength of iron. *Philos. Mag. Lett.* **82**, 141–147 (2002).
10. Roundy, D., Krenn, C. R., Cohen, M. L. & Morris Jr, J. W. The ideal strength of tungsten. *Philos. Mag. A* **81**, 1725–1747 (2001).
11. Luo, W., Roundy, D., Cohen, M. L. & Morris Jr, J. W. Ideal strength of bcc molybdenum and niobium. *Phys. Rev. B* **66**, 094110 (2002).
12. Liu, L., Zeng, X., Gou, Q., Ye, Y., Wen, Y. & Ou, P. The structural stability and mechanical properties of Cu_2MnAl and Cu_2MnIn under pressure: first-principles study. *J. Electron. Mater.* **47**, 3005–3017 (2018).
13. Alés, A. & Lanzini, F. Mechanical and thermodynamical properties of β-Cu-Al-Mn alloys along the Cu_3Al→Cu_2AlMn compositional line. *Solid State Commun.* **319**, 113980 (2020).
14. Jona, F. & Marcus, P. M. A first-principles study of the Hume-Rothery electron compounds. *J. Condens. Matter Phys.* **14**, 1275 (2002).
15. Tanaka, H., Qun, L. F., Munekata, O., Taguchi, T. & Narita, T. Elastic properties of Sn-based Pb-free solder alloys determined by ultrasonic pulse echo method. *Mater. Trans.* **46**, 1271–1273 (2005).
16. Kainuma, R., Takahashi, S. & Ishida, K. Thermoelastic martensite and shape memory effect in ductile Cu-Al-Mn alloys. *Metall. Mater. Trans. A* **27**, 2187–2195 (1996).

17. Yamaguchi, M. & Umakoshi, Y. The deformation behaviour of intermetallic superlattice compounds. *Prog. Mater. Sci.* **34**, 1–148 (1990).
18. Green, M. L., Chin, G. Y. & Vander Sande, J. B. Plastic deformation of single crystals of the Heusler alloy Cu_2MnAl. *Metall. Mater. Trans. A* **8**, 353–361 (1977).
19. Umakoshi, Y., Yamaguchi, M. & Yamane, T. Deformation and fracture behaviour of the heusler alloy Cu_2MnAl single crystals. *Acta Metall.* **32**, 649–654 (1984).
20. Otsuka, K. & Ren, X. Physical metallurgy of Ti–Ni-based shape memory alloys. *Prog. Mater. Sci.* **50**, 511–678 (2005).
21. Tanaka, Y., Himuro, Y., Kainuma, R., Sutou, Y., Omori, T. & Ishida, K. Ferrous polycrystalline shape-memory alloy showing huge superelasticity. *Science* **327**, 1488–1490 (2010).
22. Bolling, G. F. & Richman, R. H. Continual mechanical twinning: part II: standard experiments. *Acta. Metall.* **13**, 723–743 (1965).
23. Guedou, J. Y., Paliard, M. & Rieu, J. Pseudo-elasticity in ordered Fe-Al alloys. *Scr. Metall.* **10**, 631–634 (1976).
24. Yasuda, H. Y., Aoki, M., Takaoka, A. & Umakoshi, Y. Pseudoelasticity in Fe_3Ga single crystals. *Scr. Mater.* **53**, 253–257 (2005).
25. Seiner, H., Stoklasová, P., Sedlák, P., Ševčík, M., Janovská, M., Landa, M., Fukuda, T., Yamaguchi, T., & Kakeshita, T. Evolution of soft-phonon modes in Fe–Pd shape memory alloy under large elastic-like strains. *Acta Mater.* **105**, 182–188 (2016).
26. Xiao, F., Fukuda, T. & Kakeshita, T. Critical point of martensitic transformation under stress in an Fe-31.2Pd (at.%) shape memory alloy. *Philos. Mag.* **95**, 1390–1398 (2015).
27. Chen, H., Wang, Y. D., Nie, Z., Li, R., Cong, D., Liu, W., Ye, F., Liu, Y., Cao, P., Tian, F., Shen, X., Yu, R., Vitos, L., Zhang, M., Li, S., Zhang, X., Zheng, H., Mitchell, J.F. & Ren, Y. Unprecedented non-hysteretic superelasticity of [001]-oriented NiCoFeGa single crystals. *Nat. Mater.* **19**, 712–718 (2020).
28. Bonnot, E., Romero, R., Mañosa, L., Vives, E. & Planes, A. Elastocaloric effect associated with the martensitic transition in shape-memory alloys. *Phys. Rev. Lett*. **100**, 125901 (2008).
29. Kelvin, L. Mathematical and physical papers, vol. 3: elasticity, heat, electro-magnetism. Cambridge University Press, Cambridge, England, 1890.

30. Nakanishi, N. Elastic constants as they relate to lattice properties and martensite formation. *Prog. Mater. Sci* **24**, 143–265 (1980).
31. Zweben, C., Smith, W. S. & Wardle, M. W. *Test methods for fiber tensile strength, composite flexural modulus, and properties of fabric-reinforced laminates*. Composite Materials: Testing and Design (Fifth Conference), ASTM International, 1979.
